# Different doses of UV-B radiation affect pigmented potatoes’ growth and quality during the whole growth period

**DOI:** 10.3389/fpls.2023.1101172

**Published:** 2023-02-01

**Authors:** Xiaojie Wu, Bicong Chen, Jiping Xiao, Huachun Guo

**Affiliations:** College of Agronomy and Biotechnology, Yunnan Agricultural University, Kunming, Yunnan, China

**Keywords:** pigmented potatoes, UV-B, agronomic characters, nutrients, structural genes

## Abstract

**Introduction:**

UltraViolet- Biological (UV-B) plays an important role in plant growth and the formation of nutrients, especially secondary metabolites.

**Methods:**

To investigate the phenotypic changes, physiological responses, and internal genes expression of potatoes under enhanced UV-B radiation, two Yunnan native pigmented potatoes varieties named “Huaxinyangyu” and “Jianchuanhong” were exposed to different UV-B doses during whole growth duration.

**Results:**

Pearson correlation analysis and principal component analysis showed that the agronomic characters (i.e. plant height, pitch, stem diameter, and root shoot ratio) of plants treated with low dose ultraviolet (T1) did not change significantly compared with the absence of ultraviolet radiation (CK), even unit yield increased slightly; Similarly, under low UV-B radiation, photosynthetic and physiological parameters (photosynthetic rate, stomatal conductance, respiration rate, and transpiration rate) of leaves were significantly increased. In addition, low-dose UV-B treatment promoted the synthesis of tuber nutrients (e.g. phenols, chlorogenic acids, flavonoids, vitamin C, anthocyanins) and increased the expression of structural genes for anthocyanin synthesis. The number of nutrients and gene expression in tubers raised by the “Huaxinyangyu” was the highest at 84 days, and “Jianchuanhong” was the highest at 72 days. However, the higher dose of UV-B radiation (T2) will cause greater damage to the pigmented potatoes plants, making the plants reduce the yield, and significantly reduce the tuber nutrients.

**Discussion:**

This study showed that proper ultraviolet radiation will not harm pigmented potatoes, but also improve their oxidative stress tolerance, increase the structure genes expression of anthocyanins and continuously synthesize beneficial substances to improve the yield and quality of potato tubers.

## Introduction

UltraViolet- Biological radiation reaching the earth’s surface increased as the atmospheric ozone layer is destroyed (280 to 320 nm). As a result of excessive UV-B radiation, plant growth and development can be negatively affected, and their physiological metabolism can be adversely affected. Many studies have found that UV-B exposure can trigger gene activity and regulatory responses at low levels (Hara et al., 2019). Plants’ biological responses to UV-B were influenced by spectral UV-B, exposure continuity, ascendancy conditions, and species-specific genotypes (Ibañez et al, 2017; Lingwan and Masakapalli, 2022). Studying the effects of enhanced UV-B radiation on organisms and formulating effective protective measures has become a significant issue in the process of crop cultivation in the world (Cloix et al., 2012). In plant adaptation to the external environment, phenolics can eliminate free radicals, avoid membrane peroxidation, reduce plant damage caused by external stress, etc., which has an irreplaceable role for the entire ecosystem. Low-intensity laser radiation increased the content of anthocyanin, kaempferol, quercetin, and anthocyanin in leaves of Arabidopsis thaliana plants at 5 weeks of age (Dudareva et al., 2020). Under UV-B radiation, the flavonoid content in the stems and calli of *Echium orientale’s* test tube seedlings was increased, indicating that plants overcome the effects of UV-B stress by increasing the accumulation of antioxidant substances (Yildirim, 2020).

In the world, the potato (*Solanum tuberosum* L.) was the most essential noncereal food. Pigmented potatoes were rich in anthocyanins, flavonoids, chlorogenic acid, vitamin C, total phenols, and other phenolic substances (Li et al., 2021b; Navarre, 2021; Ceci et al., 2022), which have the functions of reduced cholesterol and controlled blood pressure circulation (Kaspar et al., 2011). Terpenoids and phenylpropanoids were the main types of plant secondary metabolism. The main metabolic pathway of terpenoids was the mevalonate pathway (MVA), which was related to plant nutrition level and was conducive to plant self-protection and defense against pests and diseases. The mevalonate metabolic pathway was regulated by key enzymes and rate-limited enzymes, but the expression of this enzyme gene in plants was generally low and difficult to purify (Vadali et al, 2004); Benzene C metabolites were crucial to plant development and survival, phenylalanine was produced by the shikimic acid pathway and leads to the biosynthesis of phenylalanine metabolites. Flavonoid metabolism was an important branch of phenylpropanoid metabolism, which helps plants resist abiotic and biological stresses (Dong and Lin, 2021). According to the change of heterocyclic C-ring, flavonoids were divided into chalcones, aurones, flavanones, flavones, isoflavones, dihydroflavonols, flavonols, leucoanthocyanidins, anthocyanidins and flavane 3-ol (Nakayama et al., 2019). Studies had shown that the anthocyanin biosynthesis pathway was an important branch of flavonoid metabolism. Under UV-B radiation, the genes regulated anthocyanin and flavonol biosynthesis in apple fruits had changed (Henry-Kirk et al., 2018), the key structural genes encoded proteins in regulated anthocyanin biosynthesis include *phenylalanine ammonialyase (PAL), chalcone synthase (CHS), chalcone isomerase (CHI), flavanone 3-hydroxylase (F3H), flavanone 3′,5′-hydroxylase (F3’5’H)*, *flavanone 3′-hydroxylase (F3’H)*, *dihydroflavonol 4-reductase (DFR), anthocyanin synthetase (ANS)*, and UDP glucose-flavonoid 3-O-glucosyltransferase (*UFGT)* (Jaakola, 2013). More and more evidence showed that UV-B radiation affects the quality of tuber plants by promoting gene expression of healthy compounds. Since potatoes were a highly consumed crop worldwide, a study that investigates how to increase potato contents of health-promoted compounds will be an interesting one.

Yunnan is located in the low latitude plateau, and the strong UV-B radiation is one of its unique ecological factors (Zhao and Yan, 2022). Therefore, it is necessary to study the effects of enhanced UV-B on the local characteristic crop-pigmented potatoes and its reaction mechanism after ozone destruction. It also has certain scientific research foresight to ensure the sustainable and stable development of the local pigmented potatoes industry. Scientists have conducted in-depth and systematic studies on wheat, corn, and soybean effects of enhanced UV-B radiation at home and abroad (Wagh and Nandini, 2019; Choi et al., 2022), but there were few reports on the effects of UV-B radiation on pigmented potatoes. Two pigmented potatoes cultivars with different biological characteristics were taken as the research objects in this experiment. The effects of different doses of UV-B radiation on osmoregulation substances, the specific activity of antioxidant enzymes, and the quality of underground tubers of two pigmented potatoes varieties were studied by manually adjusting the dose of UV-B radiation in indoor potted plants, and the characteristics of key structural genes regulating anthocyanin synthesis were different. This study provides a basis for further studying the mechanism of enhanced UV-B radiation on plant growth in the ecological environment. At the same time, it will provide theoretical guidance and experimental research for the introduction of pigmented potatoes and improvement of cultivation technology under the enhanced UV-B radiation in the future ecological environment.

## Materials and methods

### Plant materials and intensity of UV-B radiation

The potato materials used in the experiment were Yunnan local characteristic potato cultivars named “Huaxinyangyu” and “Jianchuanhong”, respectively. The skin of “Huaxinyangyu” was purple and the flesh was pale yellow with a purple ring, and the shape was oblate oval. The skin of “Jianchuanhong” was light red and the flesh was pale yellow with a pink ring, and the shape was reniform. Two potato plants were obtained by planted tissue culture seedlings of potatoes in pots contained a pasteurized soil sterilization mixture; the size of the basin was 11 cm*16 cm. Pots were maintained in an artificial climate box of Yunnan Agricultural University, Kunming, China (N 24°03′36.90′′, E 102°42′1.45′′m above sea level). In the absence of ultraviolet radiation (CK), under the same illumination(11000 Lux) and a photoperiod of light/darkness (12h/12 h), temperature(day/night: 20° C/18° C) and humidity(75%), the photosynthetically active radiation of one incubator was about 5 KJ m^-2^ (T1) on the same day, and the photosynthetically active radiation (PAR) of the other incubator was about 10 KJ m^-2^ (T2) on the same day.

UV-B radiation was applied artificially throughout the growth cycle of the plants. The UV-B lamp type was the FLB-5A customized by Nanjing Huaqiang Company, the power was 15W, the wavelength range was 280-320nm, and the lamp tube length was 40cm. The UV-B lamp was positioned 80 cm (T1) and 40 cm (T2) above the plants, and the height of the lamp was adjusted with the height of the plants. To perform further physiological analyses, tubers were harvested after UV-B treated for 48, 60,72, and 84 days (Since all plants died in T2 at 72 days, there was no 84 days indicator).

### Growth parameters and tuber production parameters for plants

The stem diameter and pitch of each plant were calculated from the plant height and stem diameter in each case of UV-B treatment. At the time of final harvest, after carefully removing potatoes with complete roots from the soil according to conditions, evaluate the quantity, yield, as well as root shoot ratio of each tuber. After UV-B was treated for 48 days, the above-ground plants and underground tubers grew vigorously and with the highest chlorophyll content, so the net photosynthetic rate was determined by a portable photosynthetic system (LI-6400, LI-COR, NE, USA) at UV-B treated 48 days.

### Measurement of leaves stress physiological indexes

The last four leaves of 48, 60, 72, and 84 days were used as materials to determine the stress resistance index.

The chlorophyll (Chl) was determined by the soaking method (Zulqarnain et al., 2021; Kumar et al., 2022). Weigh 0.1 g fresh leaves, add 20 mL of 95% ethanol for dark treatment until the leaves turn white, fix the volume to 25 mLwith 95% ethanol, measure the OD value under A665, A649 and A470, and calculate the chlorophyll pigmented content;

Chlorophyll fluorescence parameter (FV/FM) was measured by the PlantExplorer instrument (Li et al., 2021a). The smaller the value, the greater the damage degree;

The relative conductivity (REC) of plant leaves was measured by the conductivity method (Naz et al., 2022). Weigh 0.2 g of fresh leaves, add 10mL of deionized water to soak for 5 h, and then measure the initial conductivity. After boiled the water bath for 15min and cooled it to room temperature, count the total conductivity. The relative conductivity indicates the cell membrane permeability;

Malondialdehyde (MDA) content was determined by the thiobarbituric acid colorimetric method (Farhangi-Abriz and Torabian, 2018). Weigh 0.2 g of fresh leaves, add 10 mL of 10% trichloroacetic acid, grind to homogenate, centrifuge at 4000 rpm for 10 min, take 5 mL of supernatant, add 0.6% 4,6-dihydroxy-2-mercaptopyrimidine (TBA), cool on ice for 30 min after boiling water bath, and then centrifuge. Determine the OD value under A450, A532 and A600 respectively, and calculate the content of malondialdehyde;

The determination of superoxide dismutase (SOD) content adopts the nitrogen blue tetrazolium reduction method (Kunos et al., 2022). Weigh 0.2 g of fresh sample, add 10 mL of pH 7.8 phosphate buffer, grind to homogenate, centrifuge at 4000 rpm for 15 min, and the supernatant was the sample extract, an enzyme extract of 0.1 mL was added to reaction mixtures that contained phosphate-buffered saline (PBS), 0.75 mM p-Nitro-Blue tetrazolium chloride (NBT), 0.1 mM ethylene diamine tetraacetic acid (EDTA), 130 mM methionine, 0.02 mM riboflavin, and 0.02 mM riboflavin. A light source of 5000 1x was used to incubate and stop the reaction for 15 minutes. As controls, tubes without light or sample homogenates were analyzed in parallel. One unit of SOD activity was the amount of enzyme required to result in a 50% disruption of NBT reduction assessed at 560 nm;

The guaiacol oxidation method (Killi et al., 2020) was used for the determination of peroxidase (POD)content. Weigh 0.2 g fresh leaves, add 10 mL of pH 7 phosphate buffer and 10% PVPP, grind them to homogenate, centrifuge at 4000 rpm for 15min at 4 °C, and the supernatant was the sample extract, an extract of 20 mL of the sample was diluted in 3 mL of PBS (pH 7.0) with 20 mL of Guaiacol and 10 mL of hydrogen peroxide. 470 nm absorbance was measured 5 minutes after adding enzyme-containing sample extracts;

### Measurement of tuber nutrients

The nutrient content of potato tubers was determined by using 48, 60, 72, and 84-day fresh samples.

The total anthocyanin content (TAC) was determined by the pH differential method (Huck, 2020). Weigh 0.5 g fresh sample, add 10 mL hydrochloric acid ethanol solution, grind it into homogenate, ultrasonic at 45 °C for 30 min, centrifuge at 8000 rpm for 10 min, add 5 mL hydrochloric acid ethanol solution to the precipitation, ultrasonic at 45°C for 30 min, combine the two supernatants after centrifugation, take 1 mL anthocyanin extract and add the reaction solution with pH of 1.0 and 4.5 respectively, and determine the OD values of A_max_ and A_700_ after 1h reaction, The total anthocyanin content (mg/100g) was expressed by the content of cyanidin-3-glucoside in the sample. The specific calculation formula was:


$$TAC=\frac{\left[\left(A_{max}-A_{700}\right)-\left(A{'}_{max}-A{'}_{700}\right)\right]\times M_{W}\times D\times V\times 100}{\epsilon \times L\times m_{f}}$$

Amax and A’max were the absorbances of the reaction solution with pH of 1.0 and 4.5 at the maximum wavelength, respectively; A_700_ and A’_700_ were the absorbances of the reaction solution with pH of 1.0 and 4.5 at 700 nm; MW was the molar mass of cyanidin-3-glucoside 449.2 g/mol; D was the dilution multiple; V was the total volume of extraction solution, mL; ϵ was the average molar extinction coefficient of cyanidin-3-glucoside (26900 L/(mol · cm)); L was the width of the cuvette, taking 1 cm; mf was the fresh mass of the sampled potato, g.

The content of chlorogenic acids (CGA) was determined by ultraviolet spectrophotometry (Cao et al., 2019). Weigh 0.1 g fresh sample, add 10mL of 70% ethanol for ultrasonic extraction for 1 h, centrifuge at 8000 rpm for 6min, take the supernatant to determine its OD value in A327, fit the standard curve between chlorogenic acid and corresponding absorbance (x) according to different mass concentrations (y, mg/mL), and calculate the content of chlorogenic acid in the sample.

The content of total flavonoid (TFC) was determined by sodium nitrite aluminum nitrate colorimetry (Zhao et al., 2017). Take a 0.5 g fresh sample, extract it with 1% volume fraction of hydrochloric acid ethanol solution, extract it in an ultrasonic water bath (30° C) for 30 min, and then centrifuge it for 30 min. Take the supernatant and determine its OD value under A510. According to the standard curve fitting between rutin with different mass concentrations (y, mg/mL) and corresponding absorbance (x), the total flavone content in the sample was calculated.

For the determination of total phenol (TPC) and vitamin C (Vc) content, please refer to the instructions of the kit (Suzhou Gris Biotechnology Co., Ltd.: complete phenol g0117 F, VC g0201f).

### Extraction of total RNA and real-time fluorescence quantitative PCR (qRT-PCR) analysis

Used Trizol kit (TIANGEN BIOTECH CO., LTD.) to extract total RNA from tubers of pigmented potatoes in different growth periods. Use Evo M-MLV reverse transcription premixed kit (including gDNA removal reagent for qPCR) Ver 2 (Accurate Biology), used SYBR ^®^ Green Pro Taq HS premixed qPCR kit (including ROX) (Accurate Biology) conducts real-time fluorescent quantitative PCR reaction. StGAPDH was the reference gene. The cDNA obtained by reverse transcription was diluted 10 times and used as a template. The primers of all genes (**Table 1**) were designed with SnapGene 4.3.6 software and synthesized by Sangon Biotech (Shanghai) Co., Ltd. All primers were diluted 10 times before being used. Reaction procedure: 95 °C 2 min, 95 °C 15 sec, 60 °C 30 sec, 40 cycles, Melt Curve Stage, 2^- ΔΔ Ct^ method (Livak and Schmittgen, 2001).

**Table 1:** Primer sequences for qRT PCR reaction of structural genes.

Gene name	Forward primer (5’-3’)	Reverse primer (5’-3’)
PAL	GCCATCTAATCTCACAGCAGGAAGG	AGTTCCGAGCAGTAAGAAGCCATTG
CHS	GCCCAAGTCCAAGATCACCCATG	TCCCACCAGCAAAGCAACCTTG
CHI	TGCAGCTCCACAATGTCCATTCC	TGAACCCAAACGTAGTCGAATAGGC
F3H	GGCTTTGGTCACTGGGCAACTC	CTCGGCTTCAATCTCCTCATCTTCC
F3’5’H	ATGACGTTACGTATTAGTGAGTTGT	TCAGCAACAATAAACGTCCAAAGAT
F3’H	GGCTCGTTGTGGAATCTGACCTG	CCATTGATCTCACAGCTCTCGGATG
DFR	CCATGCTACTGTTCGTGATCCTGAG	GCTTCCTTCCACTGCCAAGTCTG
ANS	TGCCTGTTGTCCCGTATGGTTTAC	GCAAGGGTGTTTCTTTCTCGTTCTG
UFGT	TTGGGAGTGGGAGTGGAGATTCG	TCATCGCCATTAGCCATCAACAGTC
GAPDH	TGACCACTGTCCACGCCATGAC	GCTGCTCCAGTGCTGCTAGGGA

### Statistical analysis

In three independent experiments, all assays were performed. The data were means and standard errors. Two-way analyses of variance (ANOVA) were conducted using SPSS 14.0 (SPSS Inc., IL, USA) with Duncan’s new repolarization difference multiple comparison tests. P<0.05 was regarded as significant. Principal component analysis (PCA) and Pearson correlations were performed using the “ggplot2” package in R (version 3.3.4, https://CRAN.R-project.org/package=ggplot2).

## Results

### Effects of different UV-B treatments on agronomic characters and photosynthetic characteristics of pigmented potatoes

**Figure 1:**
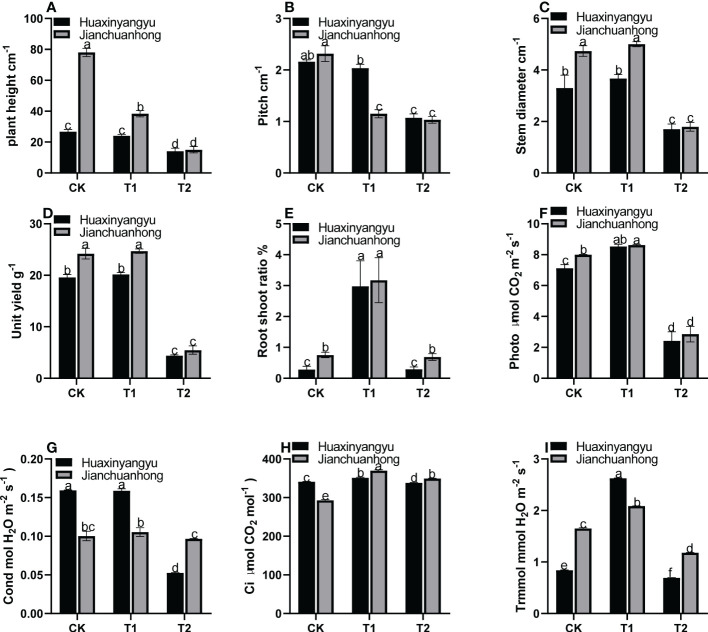
Effects of different UV-B treatments on agronomic characters and harvest indexes of “Huaxinyangyu” and “Jianchuanhong”. **(A)** Plant height, **(B)** pitch, **(C)** stem diameter, **(D)** unit yield, **(E)** root shoot ratio, **(F)** photosynthetic rate (Photo), **(G)** stomatal conductance (Cond), **(H)** respiration rate (Ci), **(I)** transpiration rate (Trmmol) (According to the Duncan test, different letters indicate a significant difference (p< 0.05) among the treatments, The error bars represent the mean ± SE. The X-axis represents processing, and the Y-axis represents height, percentage, and concentration).

As shown in **Figure 1**, the plant height and node spacing of “Jianchuanhong” in T1 and T2 decreased significantly compared with CK, while that of “Huaxinyangyu” decreased slightly. The difference between different treatments was CK>T1>T2 (**Figures 1A, B**); The stem diameter and single plant yield T1 of “Huaxinyangyu” and “Jianchuanhong” were slightly higher than CK, but the difference is not significant, while T1 and CK are significantly higher than T2 (**Figures 1C, D**); The root shoot ratio of T1 was significantly higher than that of CK and T2, and there was no significant difference between CK and T2 (**Figure 1E**); According to the photosynthetic rate (Photo), respiration rate (Ci) and transpiration rate (Trmmol) of two pigmented potatoes varieties, T1 is significantly higher than CK and T2 (**Figures 1F, H, I**), while the stomatal conductance (Cond) of T1 was slightly higher than CK (**Figure 1G**), and the difference is not significant (T1>CK>T2).

### Effects of different UV-B treatments on leaf stress physiological indexes of pigmented potatoes during the whole growth period

**Figure 2:**
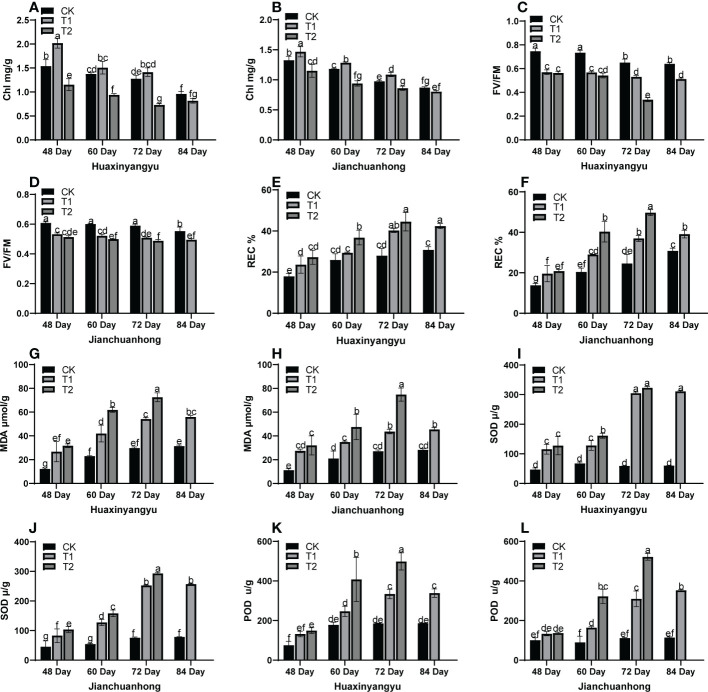
Analysis of stress physiological indexes of leaves of different UV-B treatments in the whole growth period of “Huaxinyangyu” and “Jianchuanhong”. **(A)** the chlorophyll content (Chl) of “ Huaxinyangyu “, **(B)** Chlorophyll content (Chl) of “ Jianchuanhong “, **(C)** Chlorophyll fluorescence parameter (FV/FM) of “Huaxinyangyu”, **(D)** Chlorophyll fluorescence parameter (FV/FM) of “Jianchuanhong”, **(E)** the relative permeability (REC%) of “Huaxinyangyu”, **(F)** the relative permeability (REC%) of “Jianchuanhong”, **(G)** the malondialdehyde (MDA) of “Huaxinyangyu”,**(H)** the malondialdehyde (MDA) of”Jianchuanhong”, **(I)** the superoxide dismutase (SOD) of “Huaxinyangyu”, **(J)** the superoxide dismutase (SOD) of “Jianchuanhong”,**(K)** the determination of peroxidase (POD) of “Huaxinyangyu”, **(L)** the determination of peroxidase (POD) of “Jianchuanhong”. (According to the Duncan test, different letters indicate a significant difference (p< 0.05) among the treatments. The error bars represent the mean ± SE. The x-axis represents the variety of names, and the Y axis represents compound content).

As shown in **Figure 2**, the chlorophyll (Chl) decreased with the growth period of the two kinds of pigmented potatoes, and the Chl was the highest at 48 days, and the relationship between different treatments in each growth period was T1>CK>T2 (**Figures 2A, B**). FV/FM (**Figures 2C, D**) in “Huaxinyangyu” and “Jianchuanhong” decreased significantly with the increase of UV-B radiation intensity and radiation time, and the damage degree was the highest at 72 and 84 days; The relative conductivity (REC), The malondialdehyde content (MDA), the superoxide dismutase content (SOD) and the determination of peroxidase content (POD) (**Figures 2E–G, H–L**) increased significantly with the increase of UV-B radiation intensity and radiation time, and the content was the highest at 72 and 84 days (T2>T1>CK). This result showed that with the increase of UV-B radiation time and dose, plants are under different degrees of stress (T2>T1>CK). From the chlorophyll content, it was possible to activate the metabolism of enzymes in the chlorophyll membrane under T1 treatment, thus leaded to the increase of chlorophyll content, thus increasing the photosynthetic rate, stomatal conductance, respiratory rate, and transpiration rate (**Figures 1F–I**); However, with the increase of treatment time, the enzyme activity in the chlorophyll membrane was gradually destroyed, which leads to the decrease of chlorophyll content with the growth period.

### Impacts of increased different UV-B exposure on tuber nutrients

**Figure 3:**
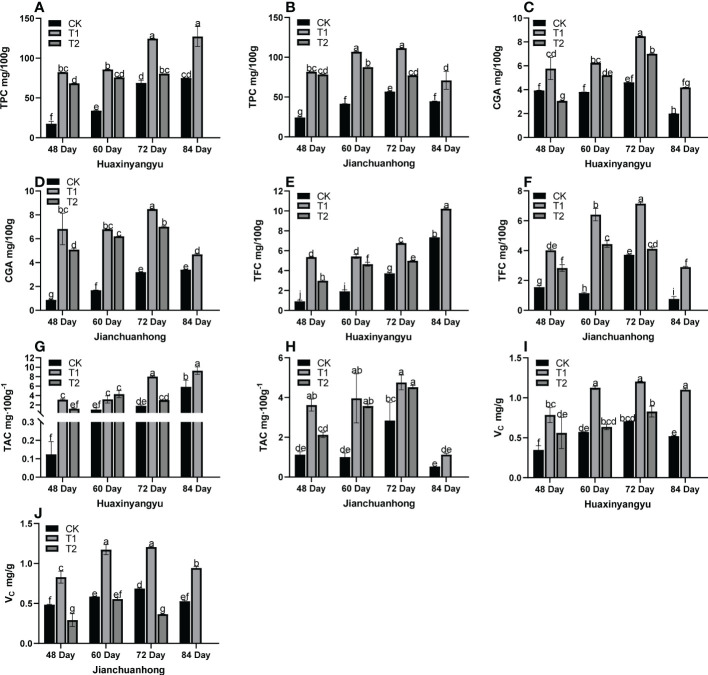
Analysis of the tuber nutrient composition of “Huaxinyangyu” and “Jianchuanhong” in the whole growth period with different UV-B treatments **(A)** the phenols content (TPC) of “ Huaxinyangyu “, **(B)** the phenols content (TPC) of “Jianchuanhong”, **(C)** the chlorogenic acids (CGA) of “Huaxinyangyu”, **(D)** the chlorogenic acids (CGA) of “Jianchuanhong”, **(E)** the flavonoids content (TFC) of”Huaxinyangyu”, **(F)** the flavonoids content (TFC) of”Jianchuanhong”, **(G)** the anthocyanins content (TAC) of “Huaxinyangyu”, **(H)** the anthocyanins content (TAC) of “Jianchuanhong”, **(I)** the vitamin C (V_C_) of “Huaxinyangyu”, **(J)** the vitamin C (V_C_) of “Jianchuanhong”, (According to the Duncan test, different letters indicate a significant difference (p< 0.05) among the treatments. The error bars represent the mean ± SE. The x-axis represents a variety of names, and the Y axis represents compound content).

Previous studies have shown that UV-B radiation may affect the secondary metabolites of potato tubers, such as anthocyanins, flavonoids, chlorogenic acid, total phenols, and vitamin C. Therefore, we analyzed the changes in nutrient composition in pigmented potatoes. As shown in **Figure 3**, the phenols (TPC), chlorogenic acids (CGA), flavonoids (TFC), anthocyanins (TAC), and V_C_ content of CK, T1, and T2 of “Jianchuanhong” increased with time-dependent effects and reached the maximum value at 72 days (**Figures 3B, D, F, H, J**), but “Huaxinyangyu” was late to 84 days (**Figures 3A, C, E, G, I**); The contents of these nutrients were significantly higher than those of CK and T2 at the time of T1 (48, 60,72,84 day).

### Correlation coefficient and principal component analysis

**Figure 4:**
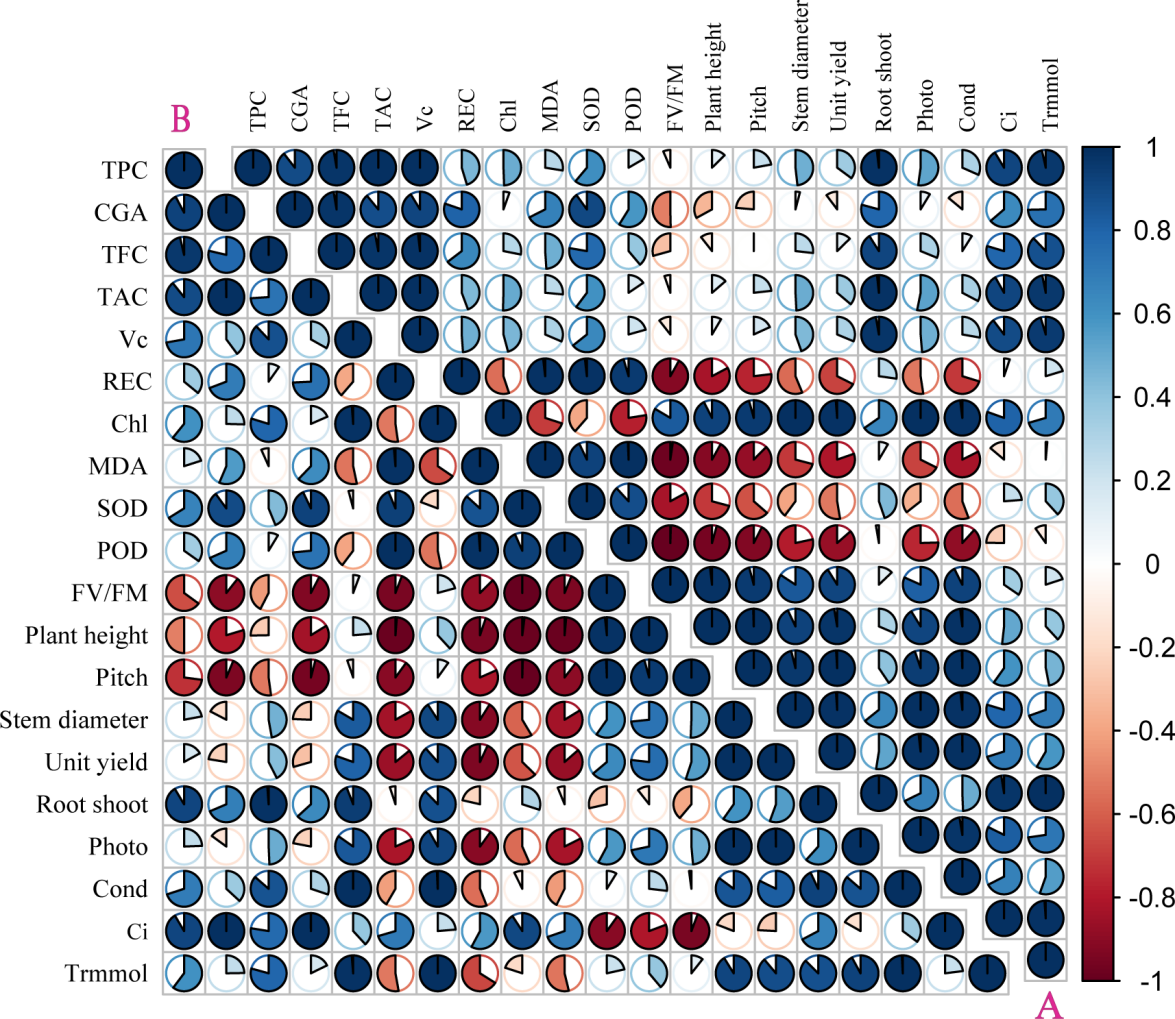
Pearson correlation analysis of different physiological of “Huaxinyangyu” and “Jianchuanhong”, **(A)** ”Huaxinyangyu”, **(B)** ”Jianchuanhong” (P<0.05). TPC, total phenol; CGA, chlorogenic acids; TFC, total flavonoid; TAC, total anthocyanin content; V_C_, vitamin **(C)**; REC, relative permeability; Chl, chlorophyll; MDA, malondialdehyde content; SOD, superoxide dismutase; POD, peroxidase; FV/FM, Chlorophyll fluorescence parameter; Photo, photosynthetic rate; Cond, stomatal conductance; Ci, respiration rate; Trmmol, transpiration rate. (According to the Duncan test, different letters indicate a significant difference (p< 0.05) among the treatments. The error bars represent the mean ± SE.).

The correlation coefficient and principal component analysis (PCA) showed that the phenotypic and physiological characteristics of the two kinds of pigmented potatoes plants treated with higher doses of UV-B decreased significantly, showing a negative correlation between phenotypic (plant height, pitch, stem diameter, yield per plant) and physiological parameters (REC, MDA, SOD, POD, FV/FM, **Figure 4**); On the contrary, low dose UV-B treatment was positively correlated with the phenotypic and physiological parameters of all plants (**Figure 4**), indicating that high dose UV-B treatment had harmful effects on the development and growth of pigmented potatoes plants. The Photo, Cond, Ci, and Trmmol of the leaves of the two kinds of pigmented potatoes were negatively correlated with REC, MDA, SOD, and POD. On the contrary, the phenotypic data were positively correlated with Chl and tuber nutrients (TPC, CGA, TFC, TAC, VC, **Figure 4**), which meant that a higher level of photosynthetic rate could increase the growth and biomass of the two kinds of pigmented potatoes (**Figure 4**).

**Figure 5:**
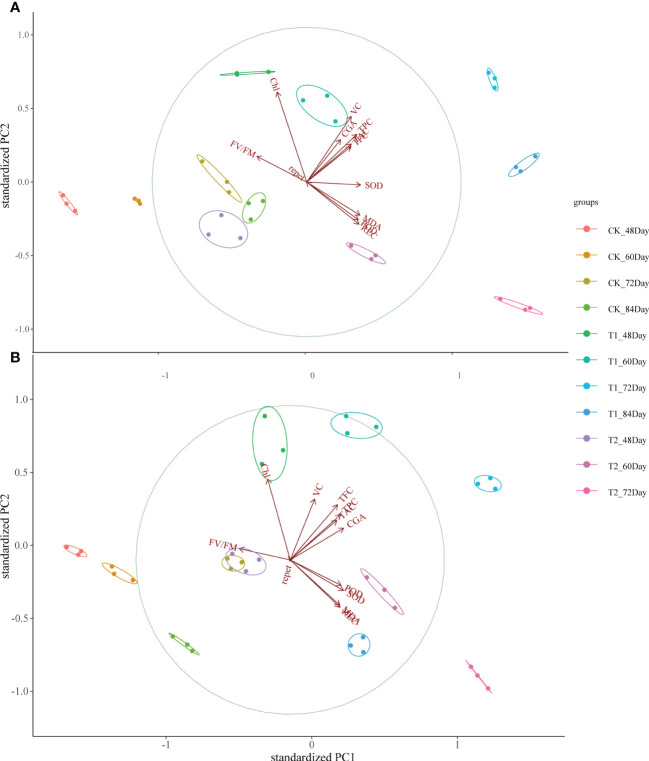
Principal component analysis (PCA) between leaf stress physiological indexes and tuber nutrient components of the whole growth period of “Huaxinyangyu” and “Jianchuanhong” plants under different UV-B treatments. **(A)** ”Huaxinyangyu” PCA, **(B)** ”Jianchuanhong” PCA. TPC, total phenol; CGA, chlorogenic acids; TFC, total flavonoid; TAC, total anthocyanin content; V_C_, vitamin C; REC, relative permeability; Chl, chlorophyll; MDA, malondialdehyde content; SOD, superoxide dismutase; POD, peroxidase; FV/FM, Chlorophyll fluorescence parameter. (According to the Duncan test, different letters indicate a significant difference (p< 0.05) among the treatments. The error bars represent the mean ± SE.).

PCA analysis of physiological parameters showed that PC1 of “Huaxinyangyu” and “Jianchuanhong” was 60.1% and 55.6% respectively. With the development of the growth period, “Huaxinyangyu” showed a positive correlation with physiological parameters, while “Jianchuanhong” showed a positive correlation with physiological parameters at the growth period of 72 days and earlier, and a negative correlation at 84 days (**Figure 5**); Both of them had a very significant positive correlation with flavonoids (**Figure 5**).

### Effects of different UV-B irradiation on the structural genes of anthocyanin biosynthesis pathway in pigmented potatoes tubers

**Figure 6:**
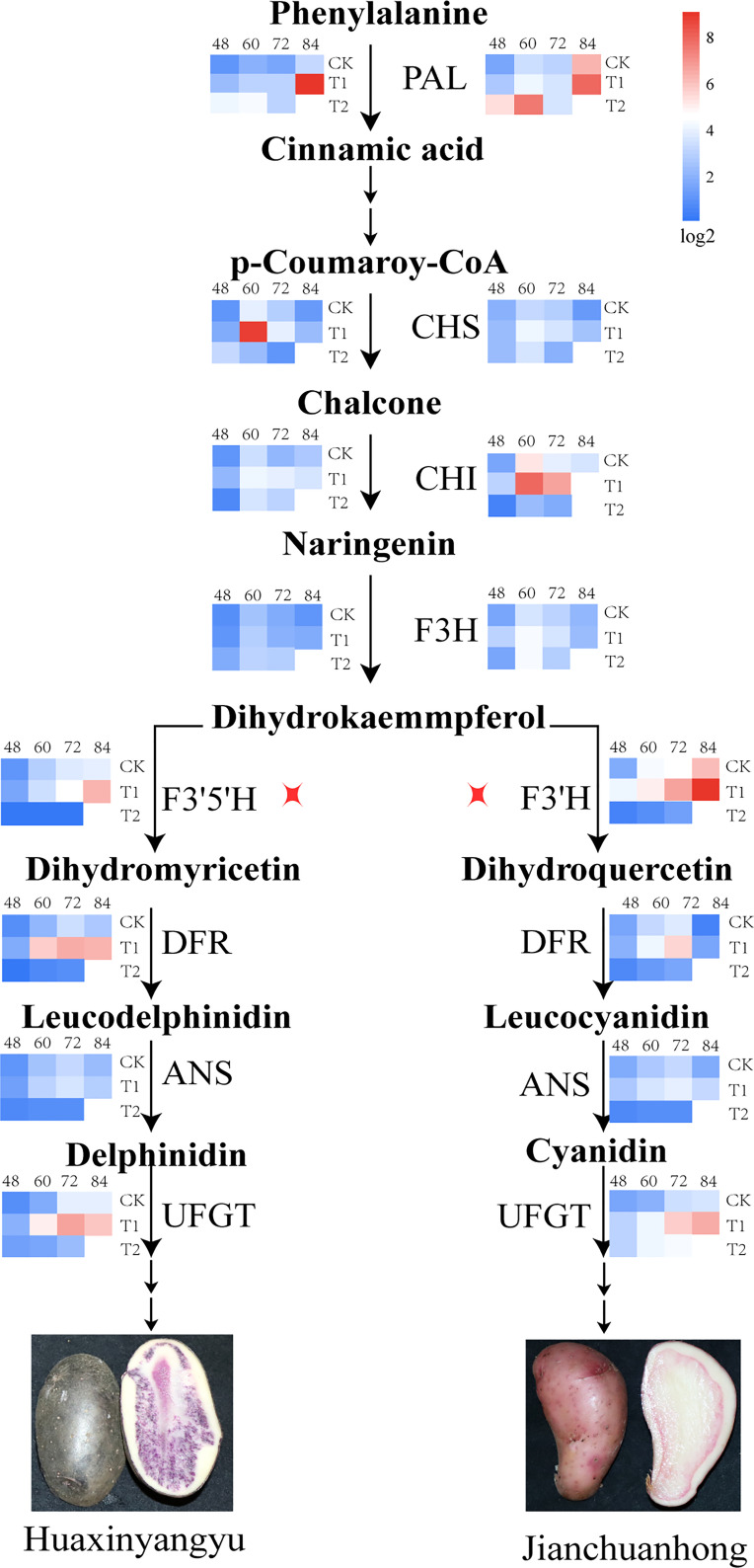
Impacts of increased different UV-B exposure on Structural Genes of Anthocyanin Synthesis Pathway in Tuber (CK, T1, and T2 were treated with different UV-B treatments, and 48, 60, 72 and 84 were the different growth stages of “Huaxinyangyu” and “Jianchuanhong”). PAL, phenylalnine ammonialyase; CHS, chalcone synthase; CHI, chalcone isomerase; F3H, flavanone 3-hydroxylase; F3’5’H, flavanone 3′,5′-hydroxylase; F3’H, flavanone 3′-hydroxylase; DFR, dihydroflavonol 4-reductase; ANS, anthocyanin synthetase; UFGT, UDP glucose-flavonoid 3-O-glucosyltransferase. According to the Duncan test, different letters indicate a significant difference (p< 0.05) among the treatments. The error bars represent the mean ± SE.).

Anthocyanin synthesis in pigmented potatoes tubers was regulated by structural genes encoding enzyme proteins (*PAL, CHS, CHI, F3H, F3’5’H, F3’H, DFR, ANS, UFGT*). Studies have found that the expression of early biosynthetic structural genes *PAL*, *CHS, CHI*, and *F3H* was the highest at 60 days, and the expression of T1 was the highest during the tuber development of “Huaxinyangyu” and “Jianchuanhong” (**Figure 6**); The expression of late biosynthetic structural genes *F3’5’H, F3’H, DFR, ANS, UFGT* was the highest at 72-84 days (there was no significant difference between 72 days and 84 days), and the expression of T1 was the highest (**Figure 6**). The expression of *PAL, CHS, CHI, F3H, DFR, ANS*, and *UFGT* in “Huaxinyangyu” was higher than that in “Jianchuanhong” (**Figure 6**), which was positively related to the content of anthocyanins.

## Discussion

### Effects of different UV-B radiation on agronomic characters and photosynthetic characteristics of potato

This study showed that the effect of UV-B radiation on plants depended on the dose of radiation. Tayebi-Meigooni et al. (2019) believe that the higher the altitude, the stronger the UV-B, and the climate conditions in high-altitude areas lead to a decrease in the cell division speed of plants and dwarf a plant, further affect the individual development of plants. With the enhancement of UV-B radiation, the plant dwarfs, the stem becomes sturdy, and photosynthesis was seriously affected, which was consistent with the results of this experiment. Stomatal opening and closing were important factors that limited the photosynthesis of plant leaves. This study showed that under T1, the Cond of pigmented potatoes leaves increased, CI and Trmmol, while under T2, it decreased, indicating that low dose UV-B radiation had a particular regulatory effect on the stomatal opening and closing of pigmented potatoes leaves. The result of UV-B radiation on the photosynthesis of pigmented potatoes leaves may be mainly caused by stomatal limiting factors (Murali and Teramura, 1987); radiation causes the peroxidation of the cell plasma membrane, affect the photosynthesis of plants, decrease the content of photosynthetic pigments in plants, and decreases the net photosynthetic rate and respiratory rate (Xie et al., 2022). However, compared with the CK, the T1 keeps the number of tubers per plant and the yield per unit area unchanged, and the root shoot ratio significantly increases. This may be because the plant obtains resources from the living environment and uses them, and then allocate the resources to the vegetative organs (roots, stems, and leaves) and reproductive organs (flowers and fruits) for the plant to grow, reproduce and resist the stress of adversity (Wang et al., 2022). Many studies have shown that the reproductive allocation of plants was closely related to the individual size of plants in both flowering and fruiting stages. The larger the particular size of plants, the more materials provided to plants for vegetative growth will be consumed, and the fewer materials provided to plants for reproduction (Widiyastuti and Subositi, 2019). Our results show that appropriate UV-B radiation has no pronounced effect on plant growth and tuber production, so the correct dose of UV-B rays won’t affect the economic impact of tubers.

### Effects of different UV-B radiation on stress indexes of potato leaves

According to our results, with the increase of UV-B rays intensity and radiation time, compared with CK, the cell membrane permeability, malondialdehyde, SOD, and POD content in plant leaves were significantly increased, Chl in leaves decreased with the growth period, but T1>CK>T2 in the whole growth period. Chu and Chen (2017) believed that high-dose UV-B radiation could damage the membrane structure and ultrastructure of chloroplasts. It leads to the decrease of Chl, which leads to the disorder of active oxygen metabolism and aggravates membrane lipid peroxidation (Harborne and Williams, 2000), triggers the generation of active oxygen, and the metabolism of active oxygen-free genes in cells was out of balance and surplus. However, a low dose of UV-B radiation for a short time can activate the metabolism of enzymes in the chloroplast membrane, which increases Chl. The excess active oxygen free radicals oxidize the unsaturated and fatty acids on the cell membrane with powerful oxidation, and the main oxidation product is malondialdehyde (MDA) (Treutter, 2006). MDA can reduce the selectivity of the membrane system for ion permeation, leading to an increase in the leakage of electrolytes in the cell, thus increasing the membrane permeability of leaf cells (Li et al., 2020), and activating the activities of antioxidant enzymes such as SOD and pod. Some scholars believe that the reduction of the movement of some antioxidant enzymes may play a role as a stimulus signal to activate other antioxidant mechanisms (Bagheri et al., 2017).

### Effects of different UV-B radiation on the development of potato tuber nutrients

Metabolite synthesis pathways were affected by UV-B, namely the mevalonate pathway and phenylpropane pathway. One of the effective ways for plants to respond to UV-B radiation injury was to synthesize flavonoids and other phenolic substances through the phenylpropanoid pathway. Such substances can shield certain UV-B radiation, and also can eliminate active oxygen free radicals in plants (Sekowski et al., 2018). Low-intensity UV-B radiation can increase the content of TPC, CGA, TFC, TAC, and V_C_. With the increase of UV-B intensity, the content of these substances decreases. The mechanism still needs to be further studied at the molecular level. Studies has shown that low flux UV-B radiation upregulates gene expression of synthetic phenolic substances (Casati and Walbot, 2003).

UV-B radiation-induced anthocyanin accumulation in medicinal plants and postharvest fruits (Ruiz et al., 2016). Potato leaves were stimulated by UV-B radiation to produce UV-B absorbing flavonoids and to activate antioxidant enzymes (Santos et al., 2006). Wild potato leaves exposed to UV-B at lower altitudes exhibited a more tremendous increase in UV-absorbing phenolic compounds (UVAC) (Ibañez et al., 2017). A significant rise in flavonoid content was also observed in underground organ radish after enhanced UV-B radiation exposure (Nithia et al., 2005). In the present study, our results showed that the TPC, CGA, TFC, TAC, and V_C_ in increased tubers with the appropriate dose of UV-B rays, and when potato plants form tubers, the radiation time increases. In “Jianchuanhong”, at 72 days of treatment, the TPC, CGA, TFC, TAC, and V_C_ of T1 were significantly higher than those of CK and T2; however, in “Huaxinyangyu”, at 84 days of treatment, the TPC, CGA, TFC, TAC, VC of T1 were significantly higher than those of CK and T2. In plants, this enzyme plays a vital role in the phenylpropanoid pathway, and phenolic compounds are synthesized by it, flavonoids and anthocyanins (Endo et al., 2022).

### Effects of different UV-B radiation on the structural genes of anthocyanin metabolism pathway in potato

Phenylpropyl metabolism was the most widely studied in plants and plays a major role in plants. Phenylpropyl metabolism provides flavonoids, and flavonoids were the material basis for plants to resist and enhance UV-B radiation. The anthocyanin synthesis pathway was an important branch of flavonoids metabolism. Anthocyanins in pigmented potatoes tubers can not only resist UV-B radiation but also have strong antioxidant properties. This study found that low-intensity UV-B radiation, as weak stress, can promote the expression of essential structural genes regulating anthocyanin synthesis. When high-intensity UV-B pressure was applied, the expression of structural genes regulating anthocyanin synthesis decreases (*PAL, CHS, CHI, F3H, F3’5’H, F3’H, DFR, ANS, UFGT*), the expression of early biosynthetic structural genes *PAL, CHS, CHI, F3H* was the highest at 60 days, and the manifestation of T1 was the highest during the tuber development of “Huaxinyangyu” and “Jianchuanhong”; The expression of late biosynthetic structural genes *F3’5’H (F3’H), DFR, ANS, UFGT* was the highest at 72-84 days (there was no significant difference between 72 days and 84 days), and the expression of T1 was the highest. The presentation of *PAL, CHS, CHI, F3H, F3’5’H, F3,H, DFR, ANS*, and *UFGT* in “Huaxinyangyu” was higher than that in “Jianchuanhong” (**Figure 6**), which was positively related to the content of anthocyanins. Early biosynthetic structural genes (*PAL, CHS, CHI, F3H*) were common flavonoid pathway genes involved in all downstream flavonoid biosynthesis. In potato tubers, the relationship between early biosynthetic structure gene expression and anthocyanin accumulation is more consistent, with high face in red and purple potatoes (Liu et al., 2015). Advanced biosynthetic structure genes include *F3’5’H, F3’H, DFR, ANS*, and *UFGT* (Stushnoff et al., 2010; Povero et al., 2011), the expression level of *IbDFR* and *IbANS* in purple sweet potato was high and was in direct proportion to the content of anthocyanins (Mano et al., 2007). In many solanaceous vegetables, the expression level of late biosynthetic structure genes was positively correlated with anthocyanin content. During the development of pepper fruit, the expressions of *CaF3’5’H, CaDFR, CaANS*, and *CaUFGT* were up-regulated at the young fruit stage and down-regulated shortly before ripening and after ripening, which corresponded to the instantaneous anthocyanin accumulation pattern of the fruit (Aza-González et al., 2013). In the pigmented potatoes tuber skin, *StF3’5’H, StF3’H* (Jung et al., 2005), *StDFR* (De Jong et al., 2003; Zhang et al., 2009), *StANS* and *StUFGT* (André et al., 2009; Jung et al., 2009; Liu et al., 2015) had high expression.

Based on the above discussion results, the UV-B radiation dose under T1 treatment can significantly improve the comprehensive resistance and quality of pigmented potatoes, and will not lead to yield decline; However, at a higher dose of UV-B radiation (T2), it will cause physiological damage; Pigmented potatoes tubers were important vegetative tissues and organs, and their anthocyanins and other antioxidants are directly related to UV-B radiation. This paper intends to use Yunnan native pigmented potatoes as materials to study the response/adaptation of potatoes to different UV-B radiation. The purpose was to preliminarily determine that it can ensure the healthy growth of potatoes under the continuous stimulation of UV-B radiation, and can also be used to maximize the accumulation of beneficial secondary metabolites of plants and improve the dose effect of comprehensive resistance, to provide scientific reference for the effective use of environmental factor response to enhance the production value of pigmented potatoes. This study shows that proper ultraviolet radiation will not harm the pigmented potatoes, but will improve their antioxidant capacity, thus increasing the expression of structural genes of antioxidant anthocyanins, and continuously synthesizing beneficial substances, to improve the quality and yield of potato tubers. Based on these results, we suggest that UV-B radiation can be appropriately increased in production and cultivation in the future to adapt to the ecological environment. However, the research on the actual production traits of potatoes, such as yield, quality, and resistance, still needs further exploration.

## Conclusion

To conclude, this study analyzed the effects of different UV-B radiation doses and radiation time on the growth and quality of pigmented potatoes varieties during the whole growth period. It was found that a relatively low UV-B radiation dose did not change the agronomic characters significantly but significantly increased photosynthetic and physiological parameters; a relatively low dose of UV-B radiation will also significantly increase the expression of nutrients in tubers and structural genes regulating anthocyanins synthesis. However, the higher dose of UV-B radiation (T2) will cause greater damage to the pigmented potatoes plants, make the plants reduced yield, and significantly reduce the tuber nutrients. This study showed that proper ultraviolet radiation will improve their oxidative stress tolerance, increase the structure genes expression of anthocyanins and continuously synthesize beneficial substances to improve the yield and quality of potato tubers. The higher dose of UV-B radiation (T2) will cause greater damage to the pigmented potatoes plants, make the plants reduced yield, and significantly reduce the tuber nutrients. This study found that the accumulation of antioxidant substances such as TPC, TFC, TAC, and V_C_ in potato tubers that enhance resistance can be significantly increased under low dose (T1) UV-B radiation. At the same time, it does not affect the normal growth of potatoes and has potential application value in future agricultural production. It provides a reference for improving the comprehensive resistance and production potential of potatoes by using the difference of UV-B radiation among regions or increasing UV-B radiation appropriately.

## Data availability statement

The original contributions presented in the study are included in the article/supplementary material. Further inquiries can be directed to the corresponding author.

## Author contributions

XW performed the experiments, analyzed the data, and wrote the manuscript. XW and BC were material growers and visualized data. JX was the material provider, and conceived and designed the project. JX and HG improved the manuscript. All authors contributed to the article and approved the submitted version.
